# Treatment Outcome of Radiotherapy Alone versus Radiochemotherapy in IE/IIE Extranodal Nasal-Type Natural Killer/T Cell Lymphoma: A Meta-Analysis

**DOI:** 10.1371/journal.pone.0106577

**Published:** 2014-09-03

**Authors:** Tianxia Deng, Cheng Zhang, Xi Zhang, Sha Wu, Yaqi Xu, Shanshan Liu, Xinghua Chen

**Affiliations:** Department of Hematology, Xinqiao Hospital, Third Military Medical University, Chongqing, China; Cardiff University, United Kingdom

## Abstract

**Background:**

Previous studies have revealed conflicting findings concerning the efficacy of radiotherapy (RT) and radiochemotherapy (RCT) in IE/IIE extranodal nasal-type natural killer/T cell lymphoma (ENKTL). In this study, we conducted a comprehensive meta-analysis to address this issue.

**Methods:**

We systematically searched PubMed, Cochrane Central Register of Controlled Trials (CENTRAL), EmBase, BISOS, Clinical Trials and some Chinese databases for relevant studies, and 2 prospective and 15 retrospective studies involving a total of 1595 patients met our inclusion criteria.

**Results:**

The meta-analysis showed no significant differences in complete remission (CR) [odds ratio (OR) 0.85, 95% confidence interval (CI) 0.42–1.72, p = 0.65], 5-year overall survival (OS) [hazard ratio (HR) 1.11, 95% CI 0.85–1.45, p = 0.43] and 5-year progression free survival (PFS) (HR 1.07, 95% CI 0.75–1.53, p = 0.70) in patients who received RT versus RCT. Furthermore, the addition of CT decreased neither systemic failure (SL) (OR 0.75, 95% CI 0.47–1.21, p = 0.24) nor locoregional failure (LF) (OR 1.17, 95% CI 0.68–2.01, p = 0. 57).

**Conclusions:**

RCT did not have an obvious advantage over RT for treating IE/IIE ENKTL.

## Introduction

Extranodal nasal-type natural killer/T cell lymphoma (ENKTL), which is also referred to as lethal midline granuloma, polymorphic malignant reticulosis, or angiocentric immunoproliferative lesions, accounts for a small fraction of non-Hodgkin lymphomas [1], [2]. It has a peculiar geographic distribution: the disease is more prevalent in Asia than it is in Western countries [3]–[5]. It is usually associated with the Epstein-Barr virus (EBV) [6]–[8] and often leads to destruction in the upper aero-digestive tract, particularly in the nasal cavity and paranasal areas [9], [10]. Additionally, tumors occur in various areas, including the skin, testis, prostate gland and orbit [11]–[14].

The optimal treatment regimen for IE/IIE ENKTL has not been completely determined. The efficacy of transplantation for IE/IIE ENKTL is still being investigated [9]. Although only a limited number of studies have been conducted, patients with a poor prognosis or advanced disease (stage III/IV) are considered the best candidates for autologous hematopoietic stem cell transplantation (HSCT) or unrelated cord blood transplantation (CBT) [15]–[18]. Therefore, for early stage (IE/IIE) ENKTL, the most suitable treatment options might be radiotherapy (RT), chemotherapy (CT) or combined therapy. Some studies have demonstrated that this tumor is highly sensitive to RT, suggesting that RT alone is a sufficient treatment regimen [19], [20]. However, other studies have suggested that RT alone has a high relapse rate, and the combination of RT and CT has been explored [21], [22]. Nevertheless, the efficacy of the additional CT was recently questioned. Numerous studies have revealed no significant differences in the treatment outcomes between RT and radiochemotherapy (RCT) for IE/IIE ENKTL [23]–[25]. Moreover, G.E. Kim et al. indicated that medical complications (such as sepsis or intractable bleeding) are more severe in patients who received RCT [24].

Although a similar study has been previously published, we used a stricter inclusion criteria and a larger sample size with a total of 1595 patients in our study [26]. Moreover, the subgroup analysis of different types of CT, countries and study designs was performed. The focus of our meta-analysis, which examined parameters including complete remission (CR), 5-year overall survival (OS), 5-year progression free survival (PFS), systemic failure (SF) and locoregional failure (LF), was to compare the effectiveness of RT with that of RCT in IE/IIE ENKTL patients.

## Materials and Methods

### Search strategy

**Figure 1:**
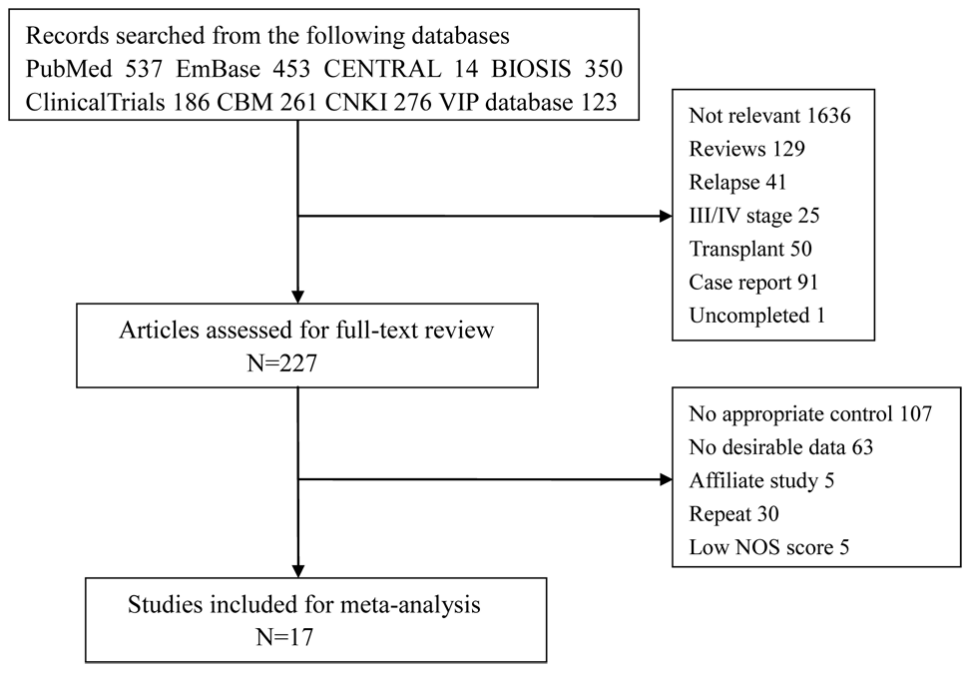
Flow chart of the identification process.

A literature search of PubMed, Cochrane Central Register of Controlled Trials (CENTRAL), EmBase, BISOS, Clinical Trials, Chinese Biological Medical literature (CBM), Chinese National Knowledge Infrastructure (CNKI) and Chinese science and technology periodical database (VIP) with the keywords ((NK/T cell lymphoma) OR (natural killer/T cell lymphoma)) AND (radiotherapy OR chemotherapy OR treatment OR outcome) was performed. The languages of the published papers were limited to English and Chinese, and only studies conducted before October 2013 were included. To include additional studies, the reference lists from the included studies were also screened. Two independent investigators conducted this search. Figure 1 depicts a flow diagram of the selection procedure.

### Inclusion Criteria

The relevant studies were carefully selected based on the following criteria: (1) patients who were confirmed as Ann Arbor stage IE or IIE ENKTL; (2) the disease was not diagnosed as a second primary carcinoma; (3) randomized controlled trials (RCT) were selected as a priori choice; otherwise, other interventional studies were included; (4) the outcomes of RT, RCT and CT were compared; (5) the article measured at least one of the following clinical indicators: CR, 5-year OS, 5-year PFS, SF or LF; (6) the latest paper was preferred if there were affiliated studies; and (7) the paper scored at least 6 based on the Newcastle-Ottawa Quality Assessment Scale (NOS) for retrospective cohort studies and at least 3 based on Jadad scale for RCTs.

### Efficacy indicators

CR was defined as the complete disappearance of all detectable clinical and radiographic evidence of disease. OS was defined as the period of time from the date of treatment to the date of death or the date of the last follow-up visit. PFS was calculated from the end date of the initial treatment to the date of the first local or distance relapse or to the date of the last follow-up or death. Any active evidence of clinical, laboratory or radiologic data in extranasal lesions was considered SF. LF was defined as persistence of the primary tumor. The indicators of CR, SF, and LF were measured by odds ratio (OR) and hazard ratio (HR) for OS and PFS.

### Data extraction

Two independent reviewers extracted the data. Discrepancies were resolved by group discussion. The primary information extracted from the studies included the first author, study design, Eastern Cooperative Oncology Group (ECOG) score and etc. The data from each paper were scrutinized. Table 1 summarizes the primary reported outcomes. Data related to the clinical outcomes, such as CR, OS, PFS, SF and LF, were also extracted.

**Table 1:** Basic characteristics of the 17 studies used in our meta-analysis.

Studies	Study design	Country	No. of patients (CT/RT/RCT)	Sex (male)	Median age	Stage (IE/IIE)	ECOG score (≤1/≥2)	B symptoms	High LDH	Primary site
HH Ma [23]	retrospective	China	0/23/41	36	44	51/13	62/2	34	9	44 patients: nasal cavity, paranasal sinus
GE Kim [24]	retrospective	Korea	0/104/39	68	46	70/34	NR	18	NR	nasal cavity/paranasal sinuses, larynx/hypopharynx, Waldeyer's ring, oral cavity/soft palate
K Kim [25]	retrospective	Korea	0/33/20	37	45	42/11	44/9	11	NR	nasal cavity, paranasal sinuses, nasopharynx, oropharynx, hypopharynx, oral cavity
A. Avilé s [28]	prospective	Mexico	116/109/202	201	NR	247/179	NR	3	40	nasal cavity, nasopharynx, paranasal sinuses, tonsils, hypopharynx, hard palate
SY Li [29]	retrospective	America	2/7/30	NR	NR	NR	NR	NR	NR	NR
JL Luo [30]	retrospective	China	2/30/98	91	NR	116/14	129/1	33	NR	NR
A Chauchet [31]	multicenter retrospective	France	8/0/8	NR	NR	10/6	NR	NR	NR	NR
YX Li [32]	retrospective	China	0/96/118	141	42	182/32	198/16	67	73	nasal cavity
YX Li [33]	retrospective	China	4/13/54	NR	NR	15/56	NR	NR	NR	nasopharynx, tonsil
MJ Huang [34]	retrospective	China	8/9/65	57	45	52/30	66/16	43	31	nasal cavity, paranasal sinuses, other parts of the upper aerodigestive tract, other sites outside nasal cavity of the upper aerodigestive tract
SJ Kim [35]	retrospective	Korea	26/0/17	30	40	24/19	37/6	NR	12	nasal cavity, nasopharynx, tonsil, oropharynx, hypopharynx, palate
K Isobe [36]	prospective	Japan	0/17/18	21	51	32/3	NR	7	7	nasal cavity, paranasal sinuses, pharynx
IWK Tham [37]	retrospective	Singapore	0/5/13	NR	NR	13/5	NR	NR	NR	nasal, Waldeyer's ring, both
CC Li [38]	retrospective	Taiwan	18/11/27	42	45	NR	NR	NR	NR	nasal area, paranasal sinuses, nasopharynx, oropharynx, pharynx, tonsils
MM Cheung [39]	retrospective	Hong Kong	0/18/61	58	53	63/16	72/7	22	20	nasal cavity, nasopharynx
V Ribrag [40]	retrospective	France	12/6/2	14	44	16/4	NR	NR	NR	nasal cavity, oropharynx, palate
YX Li [41]	retrospective	China	3/31/71	69	42	83/22	87/18	37	53	left, right, bilateral nasal cavity

CT: chemotherapy; RT: radiotherapy; RCT: radiochemotherapy; ECOG: Eastern Cooperative Oncology group; LDH: lactic dehydrogenase; NR: not reported; No.: number.

### Statistical analysis

ORs and HRs with 95% confidence intervals (CIs) were used to assess the treatment outcomes. ORs were computed for dichotomous variables by applying the Mantel-Haenszel method. HRs and the 95% CIs were computed using the Engauge Digitizer software and the method of Jayne F Tierney [27]. Statistical heterogeneity in the study was estimated by the χ^2^ test and I^2^ statistic. Values were considered to display statistically significant heterogeneity when the χ^2^ P-value was <0.1 or the I^2^ statistic was > 50%. The fixed-effects model was adopted if there was no sign of heterogeneity. Otherwise, the random-effects model was applied. Meta regression was used to locate the source of potential heterogeneity. Then, we applied the subgroup analysis to determine the potential effect on the treatment outcomes. Two-side P values less than 0.05 were considered statistically significant. To examine the publication bias, Egger text was used. All calculations were performed using STATA (version 11.0).

## Results

### Study selection results

Figure 1 shows the identification process of eligible studies. We retrieved 2,236 potential papers from the electronic databases. Of the initial collection of articles, 2,009 articles were excluded based on the title or abstract. After reading the complete text of the remaining studies, 17 articles met our inclusion criteria [23]–[25], [28]–[41]. The studies were primarily excluded because the common therapeutic measures were CT, RT or RCT alone. Some studies met the comparison criteria but had no available data. An additional 5 papers were excluded because they were affiliated studies. In addition, 30 duplicate reports and 5 papers with low NOS scores were eliminated.

### Characteristics of the final studies

17 studies were finally included. Of these, two were prospective, one was multicenter retrospective, and the remainder was retrospective. All retrospective cohort papers had an NOS score of at least 6 points (Table 2) and all prospective papers had a Jadad scale score of at least 3 points. A total of 1595 patients were enrolled and were classified as Ann Arbor stage IE or IIE ENKTL. The median age of the patients in each study varied from 40 to 53 years. Some clinical manifestations, such as B systems were also recorded. However, regrettably, some papers that included stage IIIE or IVE ENKTL lacked basic information. Based on the different extracted data, we included 3 papers by YX Li et al. that had different publication years [32], [33], [41]. The basic characteristics of the 17 studies are summarized in Table 1. The details of the CT regimen and combined therapy are listed in Table 3, and the RT information is shown in Table 4.

**Table 2:** Quality assessment of retrospective cohort studies by NOS.

Studies	Representativeness of the exposed cohort	Selection of the non-exposed cohort	Ascertainment of exposure	Outcome of interest was not present at start of study	Comparability of cohorts on the basis of the design or analysis	Assessment of outcome	Was follow-up long enough for outcomes to occur	Adequacy of follow up of cohorts	Total quality scores
HH Ma [23]	1	1	1	1	2	1	1	1	9
GE Kim [24]	1	1	1	1	2	1	1	1	9
K Kim [25]	1	1	1	1	0	1	1	1	7
SY Li [29]	1	1	1	1	0	1	1	1	7
JL Luo [30]	1	1	1	1	0	1	1	0	6
A Chauchet [31]	1	1	1	1	0	1	1	0	6
YX Li [32]	1	0	1	1	0	1	1	1	6
YX Li [33]	1	0	1	1	0	1	1	1	6
MJ Huang [34]	1	1	1	1	0	1	1	1	7
SJ Kim [35]	1	0	1	1	0	1	1	1	6
IWK Tham [37]	1	1	1	1	0	1	1	1	7
CC Li [38]	1	1	1	1	0	1	1	1	7
MM Cheung [39]	1	1	1	1	0	1	1	1	7
V Ribrag [40]	1	1	1	1	0	1	1	0	6
YX Li [41]	1	1	1	1	0	1	1	1	7

**Table 3:** Information about CT and RCT regimens.

Studies	CT regimens	RCT therapy
HH Ma [23]	CHOP, CHOP-bleo, MCOP, COPB	24 patients: RT followed by 4–6 cycles CT; 17 patients: CT followed by RT with or without CT
GE Kim [24]	CHOP, BACOP, m- BACOP	CT followed by RT
K Kim [25]	CHOP, COPBLAM-V	18 patients: 4–6 cycles CT followed by RT; 1 patients: RT followed by CT; 1 patients: concurrent RCT
A. Avilé s [28]	CEMD, ESHAP	RT followed by 6 cycles CT
SY Li [29]	CHOP	NR
JL Luo [30]	CHOP, CHOPE, DICE	73 patients: CT followed by RT; 16 patients: CT +RT(at the same time); 9 patients: RT followed by CT
A Chauchet [31]	ACVBP, CHOP, COPADM, CYVE, ESHAP, DHAP, high-dose methotrexate plus L-ASP	CT followed by RT
YX Li [32]	CHOP, CHOP-like, CEVB, DIMG	RT+4 cycles CT
YX Li [33]	CHOP, CHOP-bleo, COBVP-16, COPP	18 patients: RT followed by CT; 36 patients: CT followed by RT;
MJ Huang [34]	CHOP, ECDVP, IME	22 patients: upfront RT + CT; 43 patients: early RT plus RT+CT
SJ Kim [35]	CEOP-B	6 cycles CT followed by RT
K Isobe [36]	anthracycline-containing combination or not	NR
IWK Tham [37]	ESHAP with or without cisplatin, CHOP-M	RT firstly or after CT
CC Li [38]	CHOP, ESHAP with or without ProMACE-CytaBOM, CHOP with BDCVP, CHOP with ProMACE-CytaBOM plus combined MVP, CVPP	RT+3 cycles CT
MM Cheung [39]	ProMACE-CytaBOM, CEOP, CHOP	3–6 cycles CT followed by RT
V Ribrag [40]	CHOP, CHOP-like, COP, COP-like	CT as first-line therapy or after RT
YX Li [41]	CHOP, CHOP-bleo, COBVP-16, COPP	34 patients: RT followed by CT; 37 patients: CT followed by RT

CEMD cyclophosphamide, methotrexate, etoposide, dexamethasone; ESHAP etoposide, solumedrol, high doses of cytosine arabinoside, platinum; CHOP cyclophosphamide, doxorubicin, vincristine, prednisone; CHOPE CHOP+ etoposide; DICE dexamethasone, ifosfamide, cisplatin, etoposide; ACVBP doxorubicin, cyclophosphamide, vincristine, bleomycin, prednisone; COPADM cyclophosphamide, vincristine, prednisone, doxorubicin, methotrexate; CYVE cyclophosphamide, cytosine arabinoside, etoposide; DHAP etoposide, methylprednisolone, cytosine arabinoside, cisplatin, dexamethasone; CEVB cyclophosphamide, etoposide, vincristine, bleomycin; DIMG dexamethasone, ifosfamide, methotrexate, gemcitabine; CHOP-bleo CHOP+ bleomycin; MCOP mitoxantrone, cyclophosphamide, vincristine, prednisone; COPB cyclophosphamide, vincristine, prednisone, bleomycin; ECDVP etoposide, cyclophosphamide, doxorubicin, vincristine, prednisone; IME ifosfamide, methotrexate, etoposide; CEOP-B cyclophosphamide, epirubicin, vincristine, bleomycin, prednisone;COBVP-16 cisplatin, vincristine, bleomycin, prednisone; COPP cyclophosphamide, vincristine, procarbazine, prednisone; COPBLAM-V cyclophosphamide, vincristine, prednisone, bleomycin, procarbazine, adriamycin; CHOP-M cyclophosphamide, adriamycin, vincristine, prednisone, methotrexate; ProMACE-CytaBOM prednisone, doxorubicin, cyclophosphamide, etoposide, cytarabine, bleomycin, vincristine, methotrexate; BDCVP bleomycin, doxorubicin, cyclophosphamide, vincristine, prednisone; MVP mitoxantrone, vincristine, prednisone; CVPP cyclophosphamide, vincristine, procarbazine, prednisone; CEOP cyclophosphamide, epirubicin, vincristine, prednisone; BACOP bleomycin, doxorubicin, cyclophosphamide, vincristine, prednisone; m- BACOP methotrexate and folinic acid rescue, bleomycin, doxorubicin, cyclophosphamide, doxorubicin, vincristine, prednisone; RT: radiotherapy; CT: chemotherapy; RCT: radiochemotherapy; NR: not reported.

**Table 4:** Information about RT.

Studies	Source or Technique	Dose	Clinical target volume
HH Ma [23]	^60^Co- 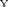 rays, 6-MV X-rays of linear accelerator	median: 54 Gy; per fraction: 1.8–2.0 Gy	bilateral nasal cavities, paranasal sinus
GE Kim [24]	^60^Co- 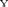 rays, 4-MV X-rays	total: 20–70 Gy; per fraction: 1.8–2.0 Gy	the involved areas with adequate margins
K Kim [25]	^60^Co- 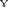 rays,4/6-MV photo beams; three-field technique or bilateral parallel-opposed fields	median:50 Gy; per fraction: 1.8/2.0 Gy	all gross lesions and sites of potential contiguous spread with adequate margins
A. Avilé s [28]	a photo beam of 6.0; IMRT, 3DCRT	a total dose of 55 Gy in 25 fractions ver 5 weeks	limited cases: bilateral nasal cavity, nasopharynx, frontal ethmoid sinus, ipsilateral maxillary; extended cases: paranasal sinus, other adjacent organ structures
SY Li [29]	NR	NR	NR
JL Luo [30]	6-MV X-rays linear accelerator; IMRT, 3DCRT	≥50 Gy: 117 patients; <50 Gy: 11 patients; per fraction: 1.8–2.0 Gy	both nasal cavity, maxillary sinus, ethmoid sinus
A Chauchet [31]	a linear accelerator with 4/6/10-MV photos	median: 40 Gy	all macroscopic lesions, paranasal sinus, nasopharynx, upper gum and palate with adequate margins
YX Li[32]	NR	primary tumor: 50 to 56 Gy; residual disease: 5 to 10 Gy; per fraction: 1.8–2.0 Gy	nasal cavity, ipsilateral maxillary sinus, bilateral ethmoid sinus, anatomically adjacent regions
YX Li[33]	6 MV linear accelerator	median: 50 Gy; per fraction: 2.0 Gy	Waldeyer ring, adjacent organs or structures with disease extension
MJ Huang [34]	^60^Co- 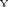 rays, 6-MV photo beams	median: 50 Gy; per fraction: 2.0 Gy	all involved area and sites of potential contiguous spread with adequate margins
SJ Kim [35]	NR	total: 44–60 Gy; per fraction: 1.8–2.0 Gy	involved-field
K Isobe [36]	^60^Co unit, 4/6/10-MV linear accelerator	median: 50 Gy	all macroscopic lesions, paranasal sinus, nasopharynx, upper gum and palate with adequate margins
IWK Tham [37]	6-MV linear accelerator	median: 50 Gy; per fraction: 1.8–2.0 Gy	gross tumor volume using diagnostic CT scans of the head and neck with a margin of 1.5–2.0 cm
CC Li [38]	6-MV linear accelerator	total: 40–50 Gy; per fraction: 1.8–2.0 Gy	involved primary area with adequate margins
MM Cheung [39]	3 fields with 2 lateral opposing photo fields and an anterior photo or electron field	median: 50 Gy; per fraction:1.5– 2.5 Gy	both nasal cavity and nasopharynx, paranasal sinus, and 1 to 2 cm beyond tumor defined by imaging scans
V Ribrag [40]	NR	total: 35–70 Gy	the initial involved areas
YX Li[41]	6/8-MV linear accelerator	median: 50 Gy; per fraction: 2.0 Gy	Stage I: nasal cavity, ipsilateral maxillary/ethmoid sinus; Stage II: extend to encompass involved paranasal tissues

RT: radiotherapy; IMRT intensity-modulated radiotherapy; 3DCRT three-dimensional conformal radiotherapy; MV megavolt; NR: not reported.

### Response to treatment

**Figure 2:**
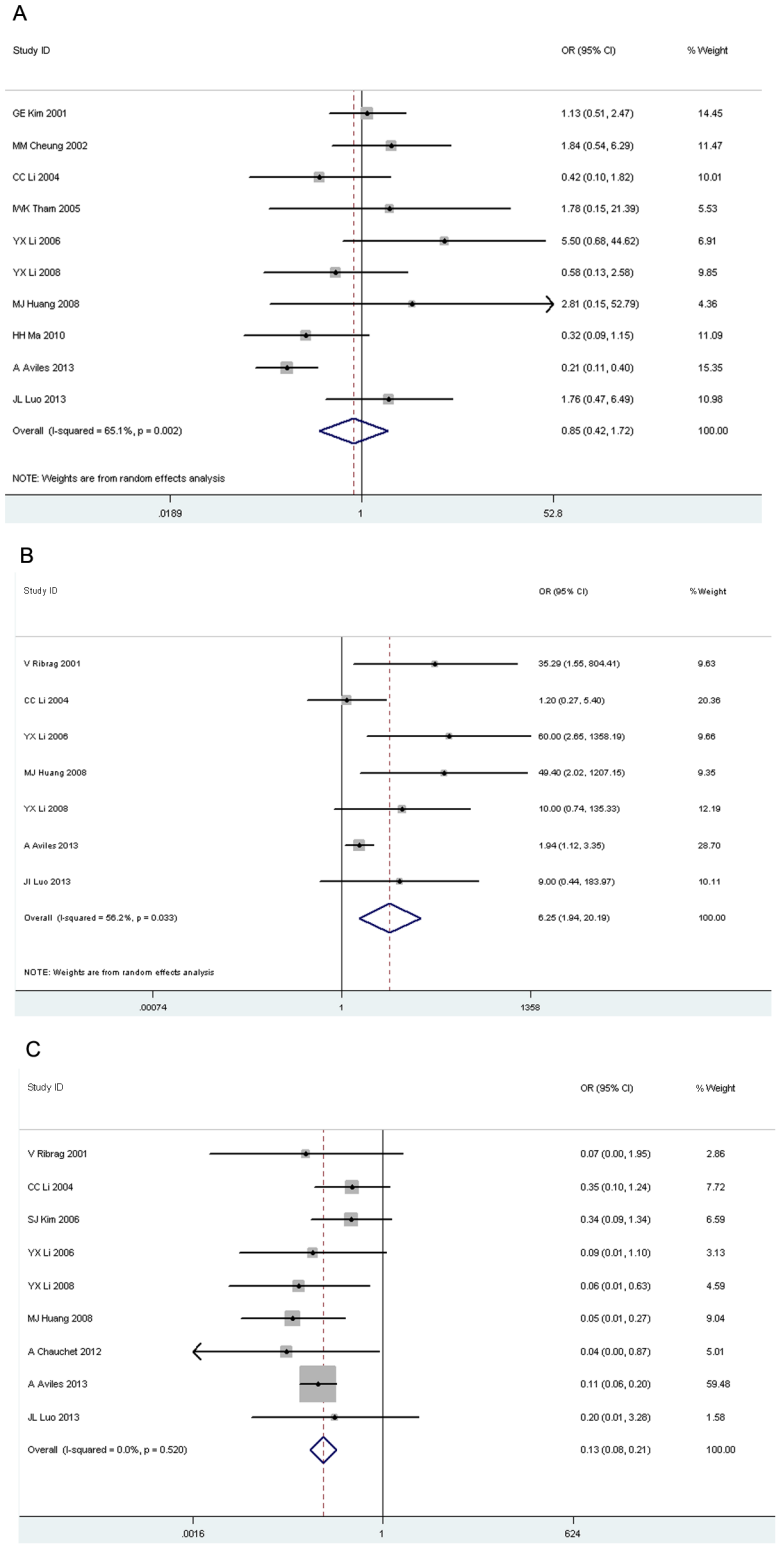
Forest plot of complete remission. A: radiotherapy versus radiochemotherapy; B: radiotherapy versus chemotherapy; C: chemotherapy versus radiochemotherapy.

CR was used to reflect the tumor's response to treatment for comparisons among RCT, RT and CT. There was no significant difference in CR between RCT and RT (OR 0.85, 95% CI 0.42–1.72, p = 0.65, Figure 2A). A higher pooled CR was found in patients who received RT or RCT compared with that of CT (OR 6.25, 95% CI 1.94–20.19, p = 0.002, Figure 2B; OR 0.13, 95% CI 0.08–0.21, p = 0.00, Figure 2C).

### Survival

**Figure 3:**
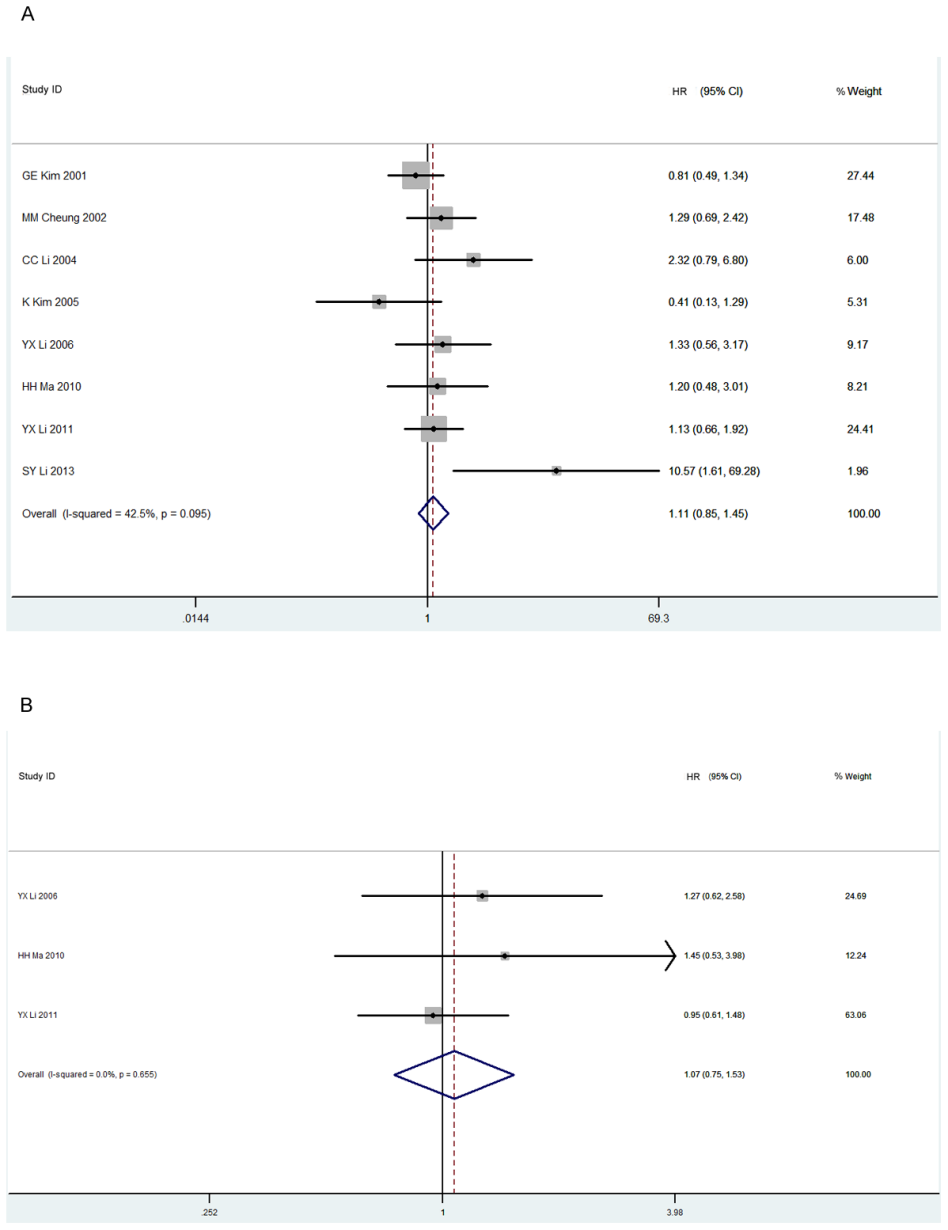
Forest plot of tumor's survival for radiotherapy versus radiochemotherapy. A: 5-year overall survival; B: 5-year progression free survival.

The 5-year OS was reported by 8 studies, in which 323 patients received RT alone and 407 patients received RCT. The addition of CT had no benefit, with a calculated HR value of 1.11 (95% CI 0.85–1.45, p = 0.43, Figure 3A). Only 3 papers were analyzed for 5-year PFS in our meta-analysis, with 150 patients in the RT group and 230 patients in the RCT group. A forest plot revealed that the treatment outcome of the RCT group failed to show any therapeutic advantage over the RT group (HR 1.07, 95% CI 0.75–1.53, p = 0.70, Figure 3B).

### Treatment failure

**Figure 4:**
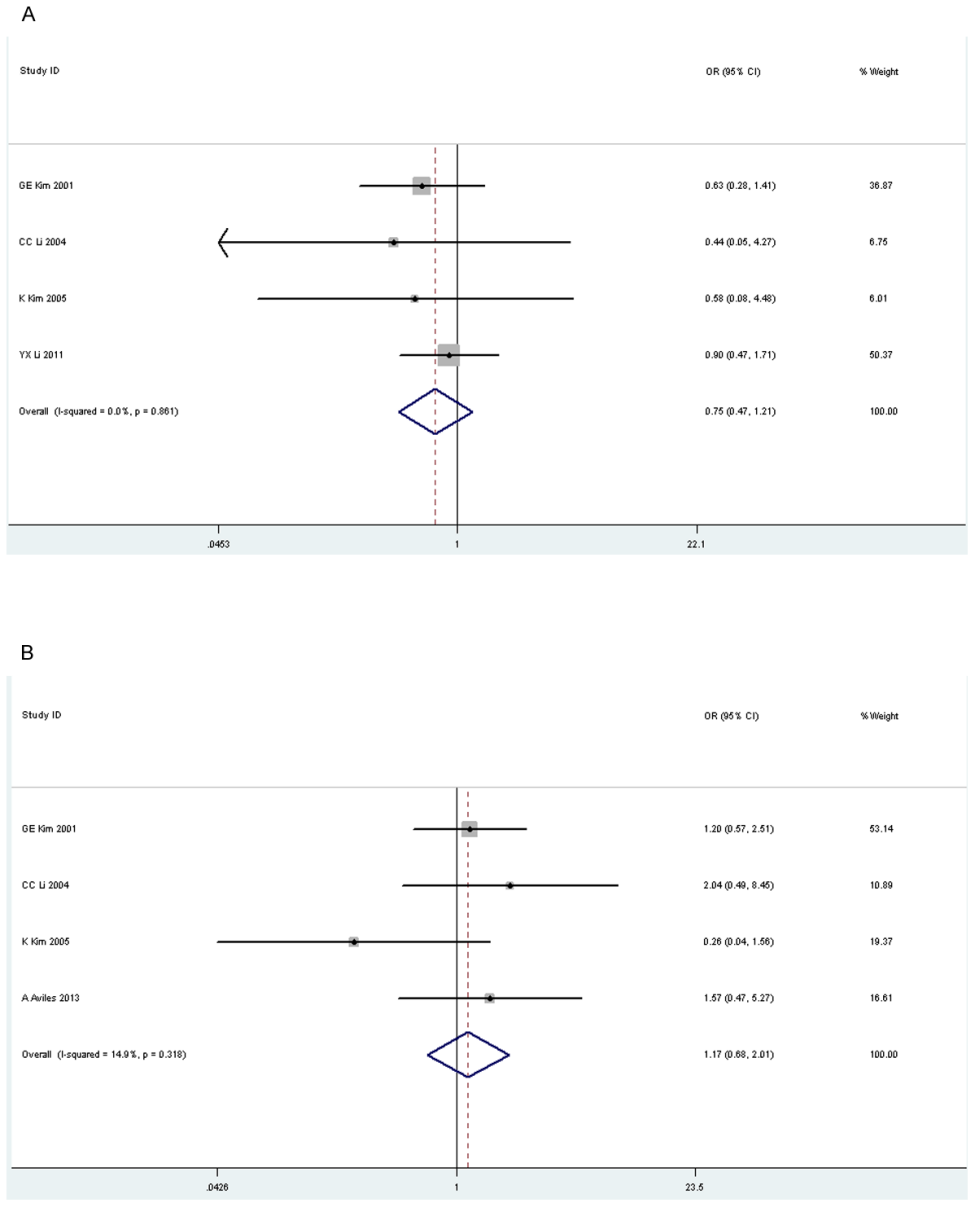
Forest plot of treatment failure for radiotherapy versus radiochemotherapy. A: systemic failure; B: locoregional failure.

As shown in Figure 4A, no significant difference was observed (OR 0.75, 95% CI 0.47–1.21, p = 0.24) in SF and LF (OR 1.17, 95% CI 0.68–2.01, p = 0.57, Figure 4B).

### Meta regression and subgroup analysis

We performed meta regression of different CT plans, countries and study designs for CR and 5-year OS. As shown in Table 5, in the group of CR for RT versus RCT, we noticed that people from Asia or not and different study designs contributed to the heterogeneity. Then, the subgroup analysis was performed. Except for the non-Asian and prospective subgroups, CR and 5-year OS showed no significant differences between RT and RCT. Nevertheless, RT showed a significant improvement in CR compared with CT, except for the non-Asian and non-China subgroups. However, in the CR for CT versus RCT group, all of the subgroups revealed that the efficacy of RCT was better than that of CT. Additionally, for the non-Asian subgroup, RCT produced a significant CR and 5-year OS benefit compared with RT or CT, and RT failed to show any advantage over CT in CR (Table 5).

**Table 5:** Subgroup analysis of CR and 5-year OS.

Indicators	Subgroup	Meta regression	OR/HR 95% CI	p value	Heterogeneity (I2, %)	p value for heterogeneity
**CR for RT versus RCT**	**CT regimen**	0.09				
	CHOP*		1.02 (0.57–1.82)	0.94	28.4	0.20
	non-CHOP		0.43 (0.06–3.10)	0.40	62.2	0.10
	**Country A**	0.02				
	Asia		1.04 (0.61–1.78)	0.87	19.7	0.27
	Non-Asia		0.21 (0.11–0.40)	0.000	-	-
	**Country B**	0.43				
	China		1.01 (0.48–2.14)	0.98	38.5	0.14
	Non-China		0.61 (0.15–2.54)	0.50	82.9	0.003
	**Study design**	0.02				
	retrospective		1.04 (0.61–1.78)	0.87	19.7	0.27
	prospective		0.21 (0.11–0.40)	0.000	-	-
**CR for RT versus CT**	**CT regimen**	0.30				
	CHOP*		10.73 (2.40–47.90)	0.002	48.0	0.09
	non-CHOP		1.94(1.12–3.35)	0.02	-	-
	**Country A**	0.67				
	Asia		9.03 (1.72–47.41)	0.009	51.7	0.08
	Non-Asia		5.44 (0.35–84.77)	0.27	69.4	0.07
	**Country B**	0.67				
	China		9.03 (1.72–47.41)	0.009	51.7	0.08
	Non-China		5.44 (0.35–84.77)	0.27	69.4	0.07
	**Study design**	0.30				
	retrospective		10.73 (2.40–47.90)	0.002	48.0	0.09
	prospective		1.94(1.12–3.35)	0.02	-	-
**CR for CT versus RCT**	**CT regimen**	0.87				
	CHOP*		0.15 (0.07–0.32)	0.000	0.0	0.50
	non-CHOP		0.13 (0.07–0.22)	0.000	28.3	0.25
	**Country A**	0.35				
	Asia		0.19 (0.09–0.38)	0.000	5.7	0.38
	Non-Asia		0.10 (0.06–0.19)	0.000	0.0	0.79
	**Country B**	0.98				
	China		0.15 (0.07–0.35)	0.000	5.0	0.38
	Non-China		0.12 (0.07–0.21)	0.000	0.0	0.41
	**Study design**	0.67				
	retrospective		0.16(0.08–0.32)	0.000	0.0	0.49
	prospective		0.11(0.06–0.20)	0.000	-	-
**5-year OS for RT versus RCT**	**CT regimen**	-				
	CHOP*		1.11(0.85–1.45)	0.428	42.5	0.095
	non-CHOP		-	-	-	-
	**Country A**	0.06				
	Asia		1.06(0.82–1.39)	0.65	8.6	0.36
	Non-Asia		10.57(1.61–69.28)	0.01	-	-
	**Country B**	0.29				
	China		1.29(0.93–1.79)	0.13	0.0	0.84
	Non-China		0.84(0.54–1.32)	0.45	76.5	0.01
	**Study design**	-				
	retrospective		1.11(0.85–1.45)	0.428	42.5	0.095
	prospective		-	-	-	-

CR: complete remission; OS: overall survival; CT: chemotherapy; RT: radiotherapy; RCT: radiochemotherapy; CHOP cyclophosphamide, doxorubicin, vincristine, prednisone; CHOP* CHOP, CHOP-like or non-CHOP.

### Toxicity and RT dose

Because toxicity and RT dose were usually reported differently, we cannot determine a pooled estimate. Grade III/IV toxicity was more frequent in the CT and RCT groups (Table 6). Controversial viewpoints regarding the appropriate RT dose were observed, as shown in Table 7.

**Table 6:** Treatment outcomes of grade III/IV toxicity.

Studies	Treatment	Total No.	No. of anemia	No. of granulocytopenia	No. of thrombocytopenia
A. Avilé s [28]	CT	116	2	6	3
	RT	109	0	0	1
	RCT	202	2	9	5
JL Luo [30]	CT	2	0	1	0
	RT	30	0	1	0
	RCT	98	5	37	6

No.: number; CT: chemotherapy; RT: radiotherapy; RCT: radiochemotherapy.

**Table 7:** Treatment outcomes for different doses of RT.

Studies	Dose/No.	Clinical indicators/No.	P value
JL Luo [29]	≥50 Gy/<50 Gy:117/11	5-year OS: 69/5	0.023
		5-year DFS: 68/5	0.027
YX Li [31]	≥50 Gy/<50 Gy:201/13	local failure: 15/2	0.592
MJ Huang [33]	≥54 Gy/<54 Gy:28/46	5-year OS: 21/21	0.019
		5-year DFS: 17/15	0.004
K Isobe [35]	≥50 Gy/<50 Gy:/9	5-year LCP: 18/5	0.13
MM Cheung [38]	≥50 Gy/<50 Gy:25/44	infield relapse: 3/12	0.4

No.: number; OS: overall survival; DFS: disease free survival; LCP: local control probability.

### Heterogeneity and publication bias

In the present study, heterogeneity existed in some comparisons. To explore the potential factors, we applied meta regression and subgroup analysis. Then the heterogeneity was decreased in most of these subgroups. Egger text was constructed to assess the publication bias of these studies. There was no evidence of publication bias in OS (p = 0.19), PFS (p = 0.14), SF (p = 0.26) and LF (p = 0.64). In the indicator of CR, a potential publication bias was observed in the RT versus CT group (p = 0.02), but not in the RT versus RCT group (p = 0.08) or the CT versus RCT group (p = 0.73).

## Discussion

Our results revealed that the clinical outcomes were similar between the RT and RCT groups, as indicated by CR, 5-year OS and 5-year PFS, although treatment of IE/IIE ENKTL with RT alone seemed to be insufficient from a clinical therapeutic standpoint. Patients who received CT alone exhibited lower CR than RT or RCT. Furthermore, RCT decreased the incidence of neither SF nor LF. Some investigations concluded that this lymphoma is sensitive to RT and resistant to CT because of the frequent expression of the multidrug resistance (MDR) genes and P-glycoprotein [23], [25], [42]. However, W Yong et al. maintained that the addition of CT to RT improved treatment outcomes, concluding that patients with nasal type NK/T cell lymphoma can be primarily treated with the CHOP regimen and local radiotherapy [43]. However, that study had some issues that deserve mention. First, the study did not include the corresponding control group of patients who were treated with RT alone. Second, the study follow-up time was only two years, but a longer observation time is necessary.

Our subgroup analysis suggested that patients from non-Asian and Asian countries usually had the opposite results. The reasons may be as follows: (1) A small number of studies were included in the non-Asian group. (2) The distribution of this disease, which is more commonly observed in Asia, is extraordinary. Thus, differences in the country of origin are the most important. (3) The design of these studies contributed to the differences. One of the non-Asian studies was prospective, while all of the Asian studies were retrospective. However, for the subgroups with different CT plans, the results did not change. Additionally, in the subgroups of prospective studies on the CR for RT versus RCT and non-China in the CR for RT versus CT, the results were different compared with those obtained in other studies. The small number of studies may have influenced this observation.

We continued to compare treatment toxicity between the groups. The results revealed that RT alone had a better effect on reducing hematologic toxicity compared with that observed in the CT or RCT groups, and it might be the best treatment option. However, the results were weakened because we were unable to determine the pooled estimates. GE Kim et al. demonstrated that the additional CT may cause medical complications, such as sepsis or intractable bleeding [24]. Additionally, we compared the appropriate RT doses. JL Luo et al. suggested that more than 50 Gy was useful for increasing the rate of 5-year OS and disease free survival (DFS) [30]. MJ Huang et al. indicated that more than 54 Gy was suitable [34]. However, other studies demonstrated that there was no difference in the RT doses regarding 5-year local control probability (LCP), local failure and infield relapse [32], [36], [39].

Most previous studies on NK/T cell lymphoma were primarily clinical studies or general reviews. To the best of our knowledge, there is only one published meta-analysis of NK/T cell lymphoma [26]. For comparison, we added 3 papers [28], [30], [36], 2 of which were prospective studies, and we excluded 3 studies with low NOS scores. We employed a more detailed process of identifying studies (Figure 1). Due to the low morbidity of the disease, we had a relatively larger sample size than that used in previous study. Additionally, we applied subgroup analysis based on the CT plans, countries and study designs. Given the uncertainty of the benefit of the additional CT and the optimal mode of therapy, we compared the treatment outcomes of RT and RCT in 17 studies. We analyzed different indicators of treatment outcome to assess the tumor response, long-term survival and treatment failure.

The limitations of our study should be acknowledged. First, most of the included studies were retrospective, though we made every effort to search for relevant studies. Therefore, our analysis may not provide strong evidence for the treatment of IE/IIE ENKTL patients. Second, most of these studies were from Asia, particularly China; therefore, our analysis may only be applicable to people in Asia. Third, due to the lack of treatment guidelines, we did not restrict the RT pattern or CHOP* group, which included the CHOP, CHOP-like, or non-CHOP regimen, and this may weaken our results. Fourth, data on the toxicity and the dose of RT were rarely available in the included studies; as a result, pooled estimates were not determined. Fifth, the limitation of language could decrease the number of included studies. Finally, the heterogeneity and publication bias may strengthen our limitations. Therefore, the results of our meta-analysis should be carefully used in clinical treatment.

## Conclusions

Compared with RT, RCT neither prolonged CR, 5-year OS or 5-year PFS nor decreased SF or LF in IE/IIE ENKTL Asian patients. However, our analysis of non-Asian patients was limited, and high quality studies are needed to identify the best therapy for IE/IIE ENKTL.

## Supporting Information

Checklist S1 **PRISMA Checklist.**
(DOC)
